# NCD-SCAN: A Digital Health Tool for Community-Based Non-Communicable Disease Risk Screening by Lady Health Workers in Pakistan

**DOI:** 10.21203/rs.3.rs-10303132/v1

**Published:** 2026-08-06

**Authors:** Zeerak Jarrar, Ghulam Kubra Rind, Saadia Sattar, Shaharyar Usman, Syed Roohan Aamir, Ambreen Gowani, Hassan Naqvi, Lijing L. Yan, Zainab Samad, Ayeesha Kamran Kamal

**Affiliations:** Aga Khan University; Aga Khan University; Aga Khan University; Aga Khan University; Aga Khan University; Aga Khan University; Aga Khan University; Duke Kunshan University; Aga Khan University; Aga Khan University

**Keywords:** Noncommunicable diseases, mHealth, Digital Health, Lady health workers, Community health workers, Low- and middle-income countries, Primary health care, Implementation research

## Abstract

**Background:**

Non-communicable diseases (NCDs) account for the majority of premature mortality in low- and middle-income countries, yet early detection remains limited within Pakistan’s primary healthcare system. Lady Health Workers (LHWs) represent a large, trusted, community-based workforce with potential to support digital NCD screening. This study assessed the feasibility, acceptability, and early implementation of NCD-SCAN, a mobile application integrating validated tools for cardiovascular, metabolic, cerebrovascular, lifestyle, and mental health risk assessment.

**Methods:**

We conducted a prospective mixed-methods implementation study in Karachi and Hyderabad, Pakistan, between June and August 2024. NCD-SCAN integrates validated instruments including RAPID, QVSFS, the Rose Angina Questionnaire, Global Adult Tobacco Survey items, the International Physical Activity Questionnaire, Refined Food Frequency Questionnaire, and the WHO-5 Well-Being Index. Formative qualitative data from 13 focus group discussions involving 128 LHWs informed application development. A separate cohort of 22 LHWs received standardized training and conducted household-based screenings. Quantitative data were analysed descriptively, while qualitative data from the formative phase and post-pilot feedback were analyzed thematically. Implementation determinants were examined using the Consolidated Framework for Implementation Research (CFIR).

**Results:**

LHWs screened 1,087 community members, with no reported data loss or technical failures. 77% of individuals were classified as medium or high risk for at least one NCD domain. High proportions of screen-positive results were observed for stroke symptoms (83.7%), poor mental well-being (64.4%), hypertension (46.3%), angina (32.8%), and diabetes risk (30.1%). LHWs reported strong community trust and perceived that the application enhanced their counselling capacity and professional credibility. Training and repeated use improved usability and confidence. Key facilitators included the application’s multilingual design, integration into existing LHW workflows, and strong community engagement, while barriers included indirect costs, limited digital access, and weak referral linkages. Suggested improvements included physician-supported referral pathways and culturally adapted multimedia content.

**Conclusion:**

Community-based digital NCD screening through Pakistan’s LHW programme is feasible and acceptable. The NCD-SCAN application supported structured risk assessment and frontline counselling; however, downstream impact will depend on strengthened referral pathways, service readiness, and financial protection mechanisms. These findings support further hybrid implementation–effectiveness evaluations to inform national digital NCD prevention strategies in low-resource settings.

## Background

Non-communicable diseases (NCDs) are the leading cause of morbidity and mortality worldwide, responsible for more than 70% of all global deaths and disproportionately affecting populations in low- and middle-income countries (LMICs).^(1)^ LMICs now face a rapid epidemiological transition driven by urbanization, demographic shifts, and persistent inequities in access to preventive and clinical care.^(2)^ The resulting rise in cardiovascular disease, diabetes, cancer, chronic respiratory disease, and mental health disorders threatens to overwhelm already strained health systems and undermine progress toward Sustainable Development Goal (SDG) target 3.4.^(3)^

Pakistan exemplifies this growing crisis. NCDs account for an estimated 58% of all deaths nationally, with ischemic heart disease, stroke, and diabetes emerging as major contributors to premature mortality and disability.^(4)^ Despite the high burden, community-level detection and management of NCDs risk factors remains limited. Access to primary care is fragmented, early screening is rare, and lifestyle counselling is inconsistently delivered. Structural constraints, including shortages of trained providers, weak referral pathways, and financial barriers, further impede timely diagnosis and continuity of care.

Parallel to this growing burden, digital health interventions have gained global traction as scalable tools for health promotion, disease monitoring, and improved patient engagement. Mobile health (mHealth) solutions, in particular, offer cost-effective and real-time communication mechanisms that support behavioral change, symptom tracking, and self-management for chronic diseases.^(5)^Evidence suggests that mHealth interventions can improve adherence, enhance health literacy, and extend the reach of primary care services especially in resource-limited settings. However, integration of mHealth into national NCD strategies in LMICs has been slow, and implementation remains inconsistent, with wide variability in usability, acceptability, and health system readiness.

In Pakistan, Community Health Workers, locally known as Lady Health Workers (LHWs) are a cornerstone of primary healthcare delivery, traditionally focused on maternal and child health.^(6)^Over time, LHWs have increasingly engaged in broader preventive and promotive health tasks and have participated in successful mHealth initiatives related to maternal health, pre-eclampsia monitoring, and immunization campaigns.^(7)^Yet, despite their substantial community presence, there is limited evidence on the use of LHW-led digital tools for comprehensive NCD risk assessment at the household level.

Given the urgent need for scalable NCD detection strategies and the untapped potential of LHWs in digital health delivery, we developed NCD-SCAN, a mobile application integrating validated screening tools for cardiovascular disease, diabetes, stroke, tobacco use, diet, physical activity, and mental well-being. This study presents the design, implementation, and pilot evaluation of NCD-SCAN in urban and peri-urban communities in Karachi and Hyderabad, assessing feasibility, acceptability, and early implementation outcomes among LHWs and the communities they serve.

## Methods

### Study Design and Setting

We conducted a prospective mixed-methods implementation study between June and August 2024 in Karachi and Hyderabad, two major urban and peri-urban centres in Pakistan. The study aimed to design, deploy, and evaluate the feasibility and acceptability of NCD-SCAN, a mobile health application for community-based screening of non-communicable disease (NCD) risk factors. The intervention was implemented through LHWs, a cadre of community-embedded health providers forming the backbone of Pakistan’s primary healthcare system.

To support a standardized implementation-focused interpretation, we used the consolidated framework for implementation research (CFIR) to allow contextualization of barriers and facilitators influencing implementation outcomes.

**Figure 1:**
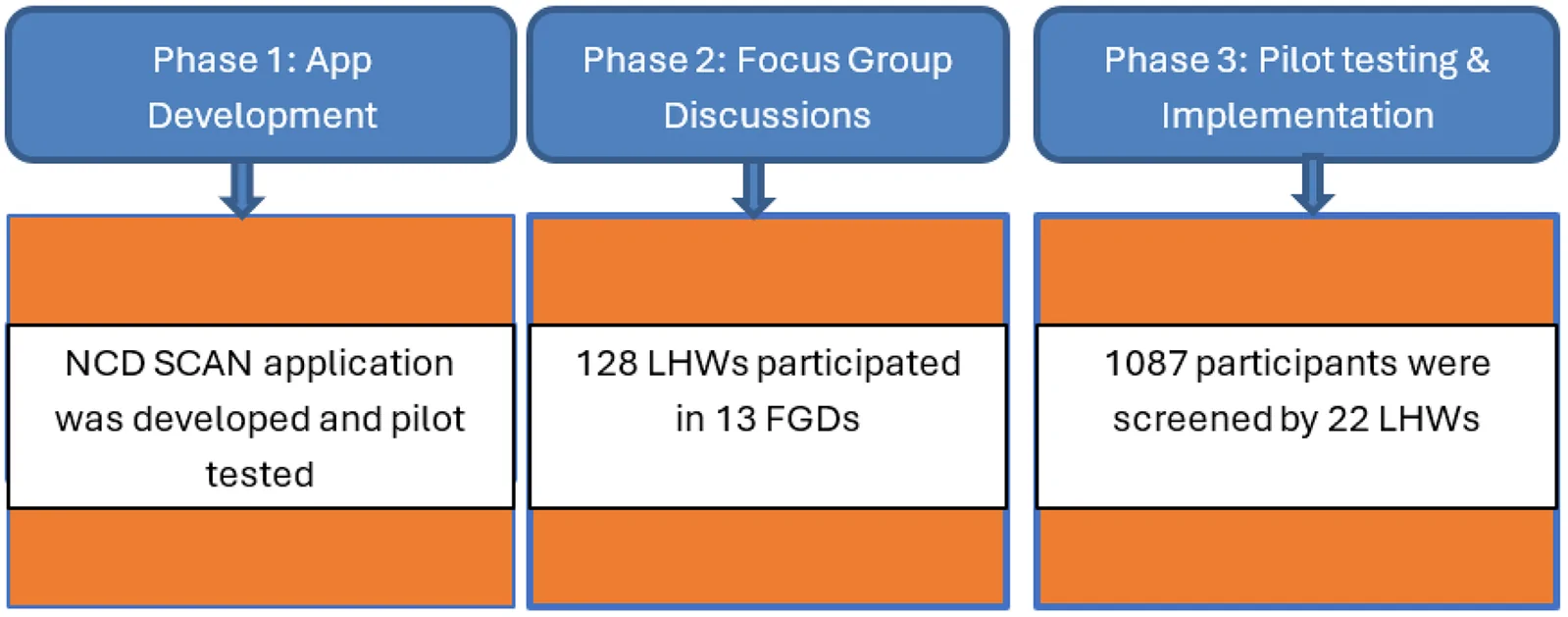
Overview of study phases including app development, LHW engagement through FGDs, and community-level pilot implementation.

The study followed three phases: development of the NCD-SCAN application, formative qualitative assessment through focus group discussions with LHWs, and pilot implementation of the application in community settings followed by post-pilot qualitative feedback, as outlined in the published study protocol. ^(27)^ (Figure 1)

A requirement analysis informed the initial architecture of the application. Using Android Studio with Java and Kotlin, we developed a multilingual (Urdu/English/Sindhi) questionnaire-based platform integrating pre-validated screening tools for NCD risk assessment. To ensure usability in low-connectivity environments, the application was designed with both online and offline data-entry capabilities. Encryption protocols and secure transfer mechanisms were incorporated to maintain data privacy and integrity.

**Figure 2:**
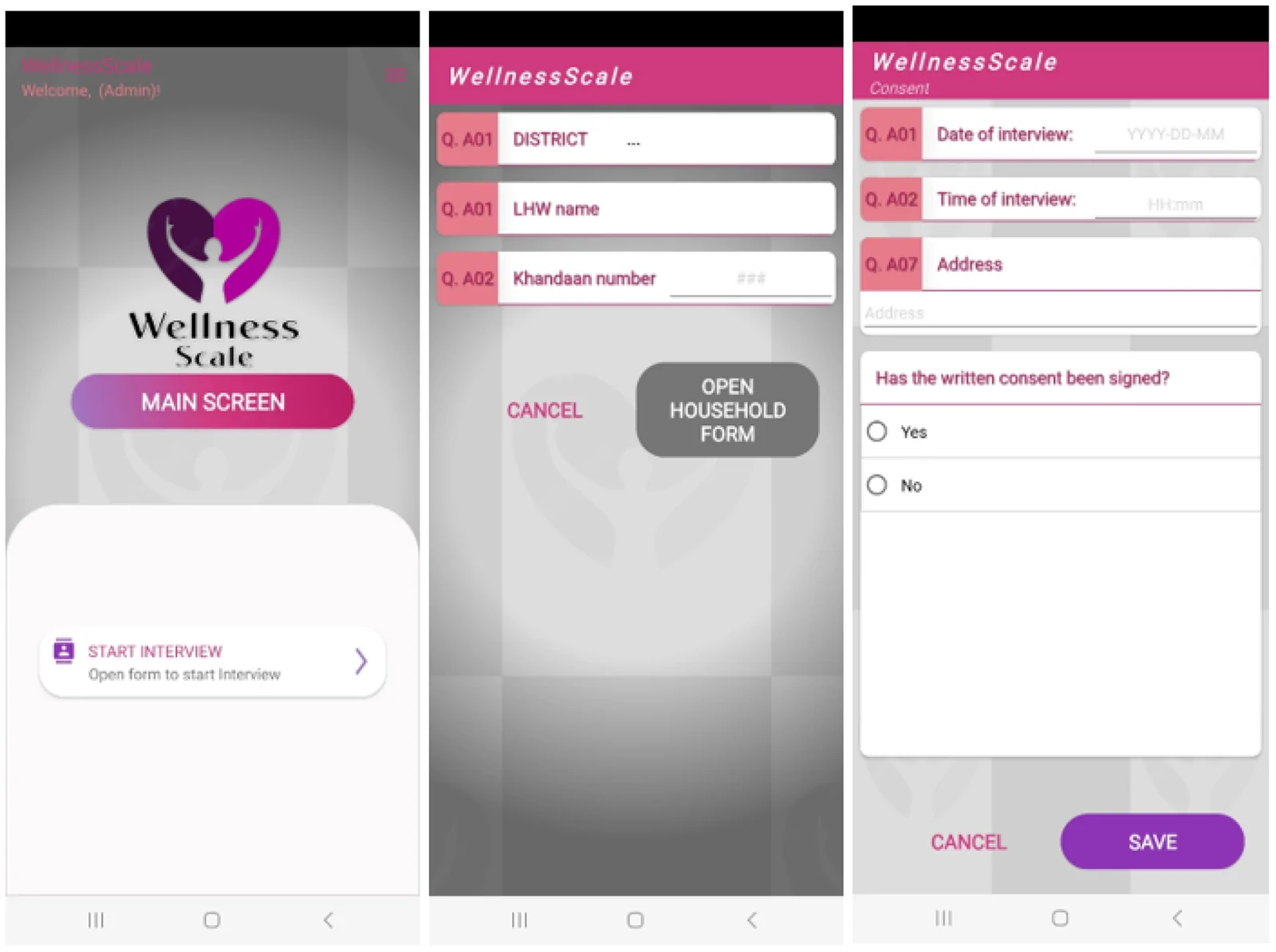
NCD-SCAN application Home screen

**Figure 3:**
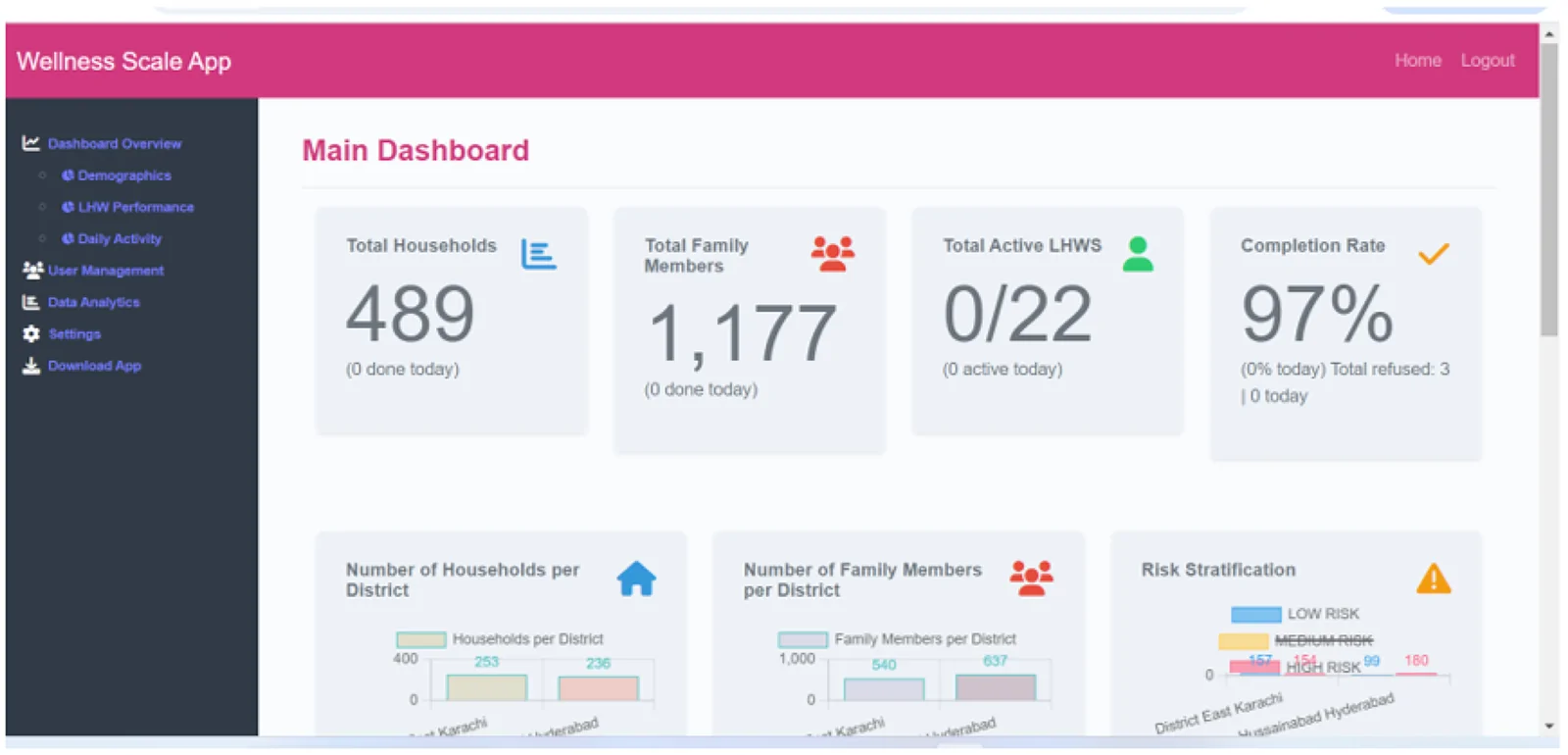
Dashboard of the NCD Scan Application

Validated instruments embedded within the application included the Rose Angina Questionnaire ^(8-11)^, the Questionnaire for Verifying Stroke-Free Status (QVSFS) ^(12)^, the Risk Assessment of Pakistani Individuals for Diabetes (RAPID) score ^(13)^, Global Adult Tobacco Survey (GATS) items ^(14)^, International Physical Activity Questionnaire (IPAQ) ^(15-17)^, Refined Food Frequency Questionnaire (RFFQ) ^(18-20)^, and the WHO-5 Well-Being Index ^(21, 22)^. Forward–backward translation methods were used to adapt all measures into Urdu. The estimated administration time for the complete toolkit was 15–20 minutes per participant. Screenshots of the NCD-SCAN application and dashboard are shown in Figures 2 and 3.

### Phase 2: Qualitative Assessment

Formative qualitative data were collected through 13 focus group discussions (FGDs) involving 128 LHWs. Eight FGDs were conducted in Karachi (District East and District West), and five were conducted in Hyderabad (Hyderabad City and Tando Jam). Sites were purposively selected to capture variation in socioeconomic context and service-delivery environments.

FGDs explored LHWs’ knowledge and perceptions of NCDs, experiences with community engagement, and views on the usability, acceptability, and feasibility of the NCD-SCAN application. The focus group discussion guide was developed as part of the study protocol, which has been published in full. ^(27)^ Discussions were conducted in Urdu or Sindhi, audio-recorded with informed consent, transcribed verbatim, translated into English, and anonymized prior to analysis.

An inductive thematic analysis approach was employed. Four researchers independently coded transcripts in pairs using line-by-line coding, followed by iterative discussions to refine codes and develop overarching themes. Themes were subsequently mapped to CFIR interpretation domains (Innovation, Outer Setting, Inner Setting, Individuals: Roles & Characteristics, and Implementation Process). Analytical rigor was strengthened through investigator triangulation, iterative code refinement, and supervisory review by a senior qualitative researcher. Thematic saturation was achieved when no new concepts emerged in successive discussions.

### Phase 3: Pilot Testing and Implementation

For the pilot phase, 22 LHWs (12 from Karachi and 10 from Hyderabad) were purposively selected. To minimize contamination between formative and implementation phases, these LHWs did not participate in the FGDs.

Participating LHWs completed a standardized three-day training programme covering core NCD concepts, risk factors, referral pathways, mental health screening, and hands-on use of the NCD-SCAN application on their own android phones. Pre- and post-training assessments were conducted to evaluate knowledge acquisition, and refresher sessions were held at three-week intervals. Printed educational materials in Urdu were provided to support community counselling.

Following training, LHWs conducted door-to-door household visits within their assigned catchment areas and screened community members using the NCD-SCAN application. Figure 1 illustrates the three phases of the study, including application development, formative focus group discussions with Lady Health Workers, and community-level pilot implementation.

Following pilot testing, brief video-recorded feedbacks were obtained from a subset of LHWs where they were asked about their experience with the application, how it assisted them in engaging community members, the public response and operational facilitators and barriers they faced. These interviews were conducted in Urdu and were subsequently transcribed and translated to English and reviewed by the research team. A brief thematic analysis of in-depth interviews (IDI) was undertaken to identify recurrent implementation-related insights that were interpreted in relation to themes identified in the formative qualitative phase of the application.

### Participants and Sampling

We used purposive sampling to recruit LHWs for the qualitative and pilot implementation phases. District Health Officers (DHOs) and Deputy District Health Officers (DDHOs) supported site identification in urban and peri-urban localities to ensure geographic and sociodemographic diversity.

A total of 128 LHWs participated in 13 focus group discussions, the details are described in Table 1. For the pilot phase, a separate cohort of 22 LHWs (12 in Karachi; 10 in Hyderabad) was selected to avoid contamination between formative and implementation components.

Community participants screened during the pilot were recruited through door-to-door visits within each LHW’s catchment area. All participants provided written informed consent.

### Data Collection and Management

LHWs used the NCD-SCAN application to enter screening data, which was stored in encrypted form on secure servers. All records were periodically exported into spreadsheets for quality checks, completeness assessment, and data cleaning. De-identified transcripts and quantitative data were stored on password-protected institutional drives accessible only to the research team.

### Quantitative Analysis

Statistical analyses were performed using Stata version 17. Continuous variables were summarized using means (SD) or medians (IQR), depending on distribution. Categorical variables were reported as frequencies and percentages. Internal consistency of screening tools was assessed using Cronbach’s alpha. Descriptive statistics were generated for all NCD risk categories (angina, stroke, diabetes, tobacco use, physical activity, diet, and mental well-being), as measured through the integrated toolkit.

## Results

### Study Participants and Screening Coverage

A total of 1,087 community participants were screened using the NCD-SCAN application by 22 trained LHWs across Karachi and Hyderabad during the pilot implementation period. All screenings were completed successfully using the mobile application, with no reported data loss or technical failures. The average duration of screening per participant was approximately 15–20 minutes.

### Sociodemographic Characteristics

The overall mean age of screened participants was 38.1 ± 14.3 years. Women constituted the majority of participants (63.8%), reflecting the household-level engagement patterns of LHWs. Educational attainment varied, with individuals having primary or secondary education representing the largest proportion of participants, while those with graduation-level education or higher comprised the smallest group. Multiple languages were represented, with Sindhi (42.2%) and Urdu (32.4%) being the most commonly reported. Detailed sociodemographic characteristics are presented in Table 2.

### Overall NCD Risk Stratification

Most participants were found to have elevated NCD risk based on screening results generated by the NCD-SCAN application. Nearly half of the screened population (47.4%) was classified as medium risk, while 30.0% were categorized as high risk for at least one NCD domain. Only 22.6% were classified as low risk, indicating that more than three-quarters of participants carried at least a moderate level of NCD risk. The distribution of overall NCD risk categories is shown in Table 3.

### Condition-Specific Screening Outcomes

Substantial variation was observed across condition-specific screening tools incorporated into the application. Hypertension risk was prominent, with 46.3% of participants classified as high risk and 23.9% as medium risk. For diabetes, 27.6% of participants were classified as medium risk and 30.1% as high risk based on the RAPID score.

**Figure 4:**
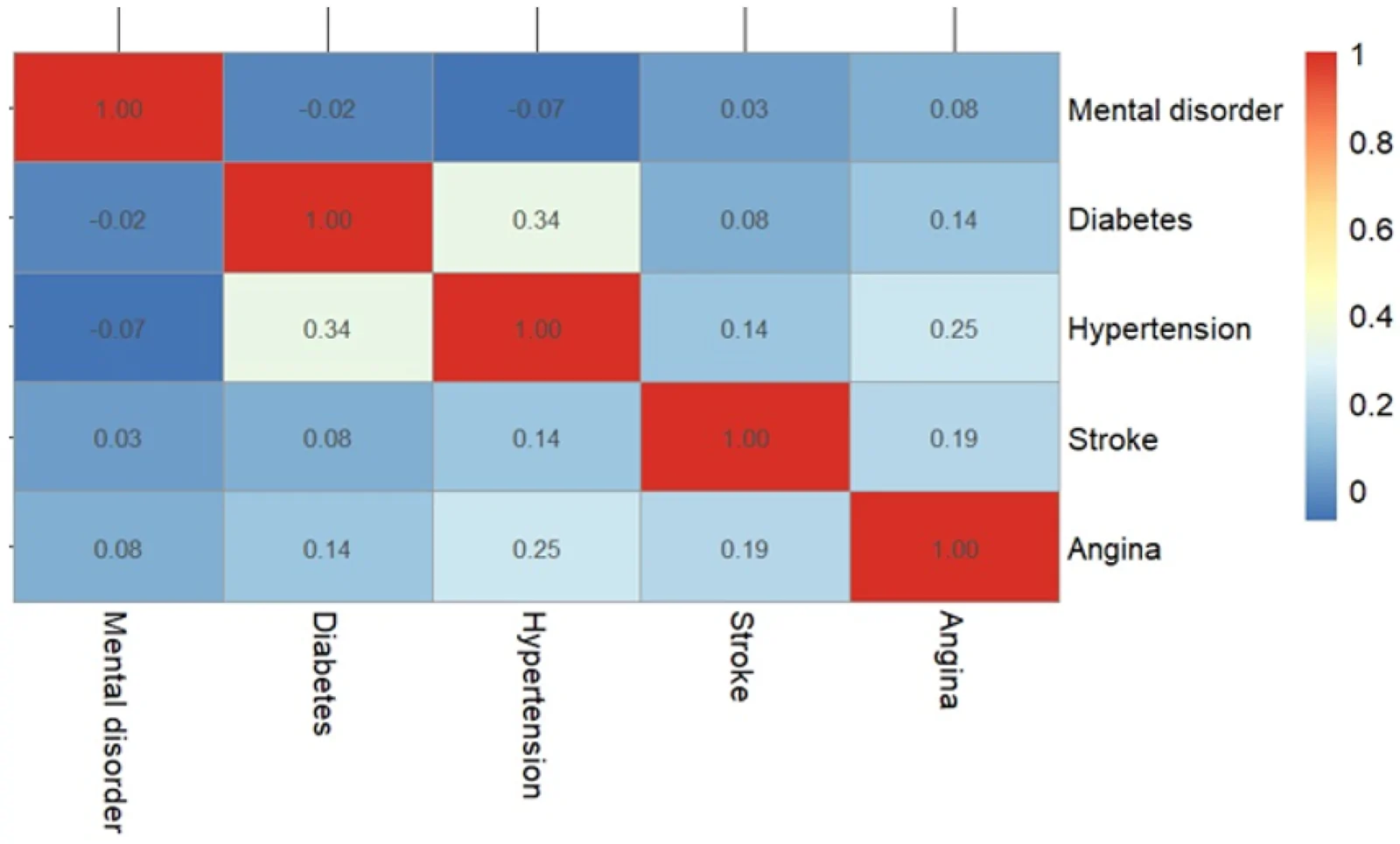
Correlational Heatmap comparing Non-communicable diseases.

Screening for stroke symptoms, assessed using the Questionnaire for Verifying Stroke-Free Status (QVSFS), identified a high proportion of screen-positive individuals, with 83.7% classified as high risk, reflecting the symptom-based and binary nature of the tool. Angina symptoms, assessed using the Rose Angina Questionnaire, were identified in 32.8% of participants. Screening for mental well-being using the WHO-5 Well-Being Index revealed a substantial burden of poor mental health, with 88.5% of medium-risk and 64.4% of high-risk participants scoring below the established well-being threshold. Condition-specific screening patterns are summarized in Figure 4.

### NCD Risk Stratification by City

Risk patterns differed modestly between Karachi and Hyderabad. In both cities, the proportion of participants classified as medium or high risk increased with age. Women constituted the majority of screened individuals across all risk categories in both locations. Physical inactivity was prevalent in both settings, particularly among medium-risk participants. Detailed stratification of NCD risk status by city, age, gender, physical activity level, and condition-specific screening outcomes is presented in Table 4.

### Correlation between different NCDs

A correlation heatmap demonstrated predominantly weak to moderate positive associations among the studied conditions. The strongest correlation was observed between hypertension and diabetes (r = 0.34), followed by hypertension with angina (r = 0.25), while stroke showed weak correlations with other cardiovascular conditions. Mental disorder exhibited negligible correlations with all variables and clustered separately, indicating limited association with the cardio-metabolic conditions in this dataset. (Figure 4)

### Qualitative Results:

13 FGDs were conducted with LHWs to explore their knowledge, perceptions, and attitudes toward non-communicable diseases (NCDs), as well as their perspectives on the usability, acceptability, and feasibility of the app prototype and change aspects based on community feedback so as to enhance user and key stakeholder uptake. The discussions were held in Urdu or Sindhi, audio-recorded with participants’ informed consent, transcribed verbatim, translated into English, and anonymized before analysis. 5 overarching themes were developed out of these FGDs: (i) Role of LHWs in the Pakistani Community; (ii) LHWs as Healthcare Providers; (iii) Perceived Limitations to the Screening App’s Utility; (iv) Suggestions for Improvement; (v) Perceived Benefits of the Application.

Findings from the formative focus group discussions informed subsequent implementation planning by identifying workflow considerations for household screening and future refinements to the application, including referral linkages and multimedia educational content.

#### Theme 1: Role of Lady Health Workers in the Pakistani Community

LHWs described a broad set of responsibilities extending beyond their traditional maternal and child health roles. They viewed themselves as educators who routinely conducted informal sessions on diet, hygiene, vaccination, and chronic disease awareness. One participant noted,

“Every second or third day we gather community women and teach them about precautions and healthy practices.”

LHWs also acted as motivators and behavioral influencers, encouraging physical activity and improved diet, with one worker sharing,

“We tell them to walk in the evenings when traffic is less.”

Their function as data collectors was well established, as they regularly documented immunization, child growth, and chronic symptoms. LHWs emphasized their bridging role between households and the primary healthcare system, stating,

“People come to us first before going to the hospital; they trust us to guide them.”

#### Theme 2: LHWs as Healthcare Providers

Participants described providing first-level counselling for a wide range of symptoms, including minor ailments and early indicators of chronic disease. One LHW explained,

“We provide first aid and then refer them.”

Many expressed a strong work ethic and deep personal commitment to their communities, stating,

“We are available to people even at 2:00 am.”

Despite this, they recognized limits to their authority, noting that they could not prescribe medication:

“We cannot tell what specific problem a person has; we only guide and refer.”

Several LHWs reported an increasing burden of NCDs, particularly diabetes and breast cancer, with one observing,

“Every second person has diabetes now, even young girls.”

#### Theme 3: Perceived Limitations to the Screening App’s Utility

While LHWs welcomed the app, they identified several barriers to its effective use. Financial constraints were a major concern, as participants felt community members often avoided follow-up due to cost:

“I don’t have money for food; how can I afford a checkup?”

Technological barriers, such as a lack of smartphones, poor internet access, and shared mobile data packages, were also widely reported. One LHW explained,

“The internet package I buy is finished by my children watching cartoons.”

Distrust in government facilities further hindered referral uptake, with LHWs noting,

“People don’t trust services at government hospitals, so they avoid referrals.”

Additional concerns included difficulty engaging men during daytime visits and increased workload due to the addition of digital tasks.

#### Theme 4: Suggestions for Improvement

LHWs recommended linking the app to physician consultation or remote support to increase acceptability, stating,

“If we could directly link patients to doctors, it would make a big difference.”

They suggested incorporating an online referral system to streamline follow-up and adding educational videos to support community understanding:

“Videos explaining diseases and treatment would be very helpful.”

Several workers emphasized the importance of continuous training and support.

#### Theme 5: Perceived Benefits of the Application

Overall, LHWs expressed strong enthusiasm for the NCD-SCAN application. They appreciated the use of Urdu, which enhanced usability, and felt the tool strengthened their credibility:

“This app will help us explain risks on the spot, and people will trust us more.”

Many believed that the app could reduce unnecessary clinic visits and improve health decision-making:

“People will not have to go somewhere else; they will know their risk immediately.”

### CFIR-guided implementation determinants:

Across intervention characteristics, the multilingual interface and offline functionality enhanced the adaptability of the application. The incorporation of validated screening questionnaires further strengthened the intervention’s design quality and perceived evidence strength. However, the relatively long duration of tool administration (15–20 minutes), costs associated with digital infrastructure, and the inability of LHWs to diagnose specific conditions increased perceived complexity and cost, while reducing the relative advantage of the intervention beyond screening.

In the outer setting, mixed patterns were found. Although the high unmet burden of NCDs and presence of national action plans to tackle NCDs align with patient needs and external policies, financial limitations to following up on referrals and poor infrastructure for healthcare linkages/referrals can impede the successful implementation of this intervention. Within the inner setting, LHWs reported limited linkage to physicians and described their role as lacking decision-making authority highlighting gaps in networks and organization. In contrast, the longstanding culture of the LHW programme, strong service ethic among LHWs, district-level support, and training-associated improvements in confidence were identified as facilitators of implementation readiness and feasibility.

Moreover, characteristics of the LHWs-including their awareness of the growing NCD burden, improved self-efficacy reflected by increased confidence in counseling following training, and their role as trusted providers within the community-were identified as facilitators of the intervention. In addition, multiphase and structured planning, high implementation fidelity with no data loss, strong engagement of community members, and active reflection and evaluation by LHWs through suggestions for improvement functioned as key facilitators within the implementation process. (Table 5)

#### Feasibility of Digital Screening

The pilot demonstrated high operational feasibility across both urban and peri-urban settings. All screening records were successfully captured using the application’s offline functionality and later synchronized without data loss. Pre- and post-training assessments indicated improved LHW knowledge across major NCD domains and confidence in using the digital tool, supporting the feasibility of task-shifted digital NCD screening in community settings.

### Post-pilot feedback from Lady health workers:

A brief thematic analysis of post-pilot feedback identified themes that largely mirrored those observed during the formative qualitative phase. (Table 6) LHWs reported initial difficulty in using the application; however, repeated hands-on training, peer learning, and continued trainer support facilitated rapid familiarity and confidence. Consistent with formative findings, the established trust of LHWs within their communities emerged as a key facilitator for participant engagement. The application also supported real-time counselling, with LHWs noting that explaining disease risks and preventive strategies prompted immediate interest among community members in adopting healthier behaviors. Key operational barriers included challenges in asking culturally sensitive questions and limited access to male participants, who were often unavailable during routine daytime visits due to work commitments.

## Discussion

In this mixed-methods study, we found that community-based digital screening for non-communicable diseases (NCDs) using the application NCD-SCAN is feasible and acceptable within Pakistan’s Lady Health Worker programme. Qualitative findings highlighted the central role of LHWs as trusted community figures who bridge households and the formal health system. LHWs perceived that the application enhanced community interactions by enabling structured risk communication and counselling, while peer and supervisory support facilitated implementation. Key barriers included the absence of functional referral pathways and reluctance of community members to seek follow-up care because of financial constraints. Quantitatively, a substantial proportion of screened participants were classified as medium (n = 516; 47.4%) or high risk (n = 325; 30.0%). The proportion of individuals classified as medium or high risk for NCDs increased with advancing age, and physical inactivity was prevalent within both groups.

Our findings reflect broader epidemiological transitions described across low- and middle-income countries (LMICs), where cardiovascular disease, diabetes, hypertension, and mental health conditions increasingly contribute to premature mortality and disability. ^(1, 4)^In Pakistan, rising cardiometabolic risk and insufficient early detection mechanisms underscore the need for scalable primary prevention approaches^.(5, 6)^

The integration of the NCD-SCAN application into the routine LHW workflow demonstrates how digital task-sharing can strengthen frontline NCD screening and counselling. A recent comprehensive review by *Xiong et al.*, which synthesized evidence from 52 studies evaluating mHealth interventions for NCDs in low- and middle-income countries, reported favorable implementation outcomes, particularly for user experience, access to health information, and capacity building among frontline providers.^(30)^ In line with these findings, LHWs in our study completed household screenings with high fidelity, experienced minimal technical difficulty, and demonstrated significant improvements in NCD-related knowledge following structured training. These observations are also consistent with evidence from LMIC settings showing that community health workers (CHWs) can effectively use digital platforms for data collection, symptom screening, health promotion, and decision support.^(7, 23, 24)^

Digital health initiatives previously implemented among LHWs in Pakistan, including maternal health, pre-eclampsia monitoring, mental health evaluation, and immunization applications- have shown similar patterns of feasibility, engagement, and improved service delivery.^(24, 25)^Notably, a recent study conducted in rural Sindh evaluated the feasibility of *mPareshan*, a mobile mental health application that supported LHWs in screening community members for depression and anxiety using standardized assessment scores. Using a comparable qualitative study design with focus group discussions, the authors reported promising uptake and acceptability of the application, while also identifying system-level challenges related to workload and follow-up care. ^(31)^ These findings demonstrate that mHealth interventions are acceptable across diverse health domains in Pakistan. By extending this functionality to NCDs, our study fills an important evidence gap in Pakistan’s digital health ecosystem.

Qualitative findings further illuminate the relational dynamics that underpin the successful deployment of community-based digital interventions. LHWs described themselves as trusted educators, motivators, and intermediaries, an identity shaped by longstanding embeddedness within households and communities. This relational proximity appeared to enhance community receptivity to digital screening and strengthened the credibility of risk information communicated through the app. These dynamics align with international analyses highlighting that CHW trust and community presence are essential determinants of digital tool uptake in LMICs. ^(7, 23, 26)^

Moreover, CFIR-guided analysis identified several individual- and process-level facilitators. The LHWs showed strong knowledge of the NCD burden, actively reflected on potential improvements to the application and exhibited a strong professional identity and service ethic. These attributes likely supported uptake and reinforced community trust, consistent with previous literature. Evidence shows that community health workers are increasingly involved in raising awareness of noncommunicable diseases across diverse community settings, including home visits, group gatherings and places of worship. ^(36)^ They also play a critical role in addressing context specific misconceptions regarding key non communicable diseases, such as myths surrounding cervical cancer screening. ^(32)^

Despite these facilitators, weak referral pathways and the restricted scope of LHW roles, largely limited to guidance and referral rather than diagnosis, emerged as important barriers. This finding highlights a mismatch between early community-based detection and the downstream system capacity required for confirmatory evaluation and management^.(37)^ Although studies from LMICs have shown that community-based and other non-physician healthcare workers can support referral, follow-up and in some settings, protocol-driven medication management, these roles remain context-specific and variably implemented. ^(33,34,35)^ Taken together, these findings suggest that stronger integration of LHW-led screening with referral and physician support may improve distribution of workload within the healthcare system, while also underscoring the need for clearer delineation and formalization of community health worker roles.

In addition, financial constraints emerged as a dominant barrier to care-seeking, with many households unable to pursue recommended consultations, diagnostic tests, or medications. This mirrors global evidence that digital tools often improve early detection but cannot independently address structural constraints such as out-of-pocket expenditures, fragmented referral pathways, and health-system inequities.^(27)^ Concerns regarding the quality and reliability of government primary health facilities—particularly shortages of qualified staff and inconsistent service readiness further reduced the likelihood of follow-up. Similar health system limitations have been documented in Pakistan and other LMICs implementing digital health interventions.^(3, 25)^

Technological and social barriers also affected adoption. Limited smartphone availability, inconsistent network connectivity, and the financial burden of mobile data packages complicated implementation, particularly in low-income households. These constraints are well recognized in the literature assessing mHealth deployment in resource-constrained settings.^(2, 7, 25)^Gendered patterns of access were also important: because LHWs predominantly interact with women during daytime visits, adult men, a key demographic for cardiovascular risk, were underrepresented in screenings. These challenges highlight the need for implementation models that account for digital divides, gender norms, and household decision-making structures.

Despite these limitations, LHWs offered clear and practical recommendations for strengthening the intervention. Their call for integrating digital referrals, teleconsultations, and audiovisual educational content aligns with global best practices for enhancing engagement and comprehension in low-literacy settings.^(23)^ The strong endorsement of Urdu and Sindhi language content further reinforces the importance of culturally adapted interfaces in digital health tools. The high prevalence of poor mental well-being detected through WHO-5 screening also indicates an urgent need to embed mental health screening and referral within Pakistan’s primary care and NCD frameworks, echoing WHO recommendations and regional analyses.^(28)^

### Strengths and Limitations

This study has several strengths. It employed a mixed-methods design, integrating quantitative screening data with qualitative insights from LHWs, which allowed for a comprehensive assessment of feasibility, acceptability, and early implementation dynamics. The intervention was evaluated under real-world conditions across diverse urban and peri-urban settings, enhancing the practical relevance of the findings. In addition, the NCD-SCAN application incorporated multiple validated screening instruments adapted to the local context, enabling a broad assessment of cardiometabolic, cerebrovascular, lifestyle, and mental health risk factors.

Several limitations should also be considered when interpreting the findings. First, screening outcomes were not linked to confirmatory clinical diagnoses or longitudinal follow-up, limiting the ability to assess downstream clinical effectiveness or health outcomes. As a result, risk classifications should be interpreted as screen-positive indicators rather than diagnoses. Second, the use of symptom-based tools such as the Questionnaire for Verifying Stroke-Free Status and the Rose Angina Questionnaire may have led to overestimation of disease prevalence at the population level, despite prior validation of these instruments in South Asian settings.^(12, 29)^

Third, the study was conducted in two urban and peri-urban districts and may not fully capture implementation challenges in rural or remote areas, where digital infrastructure, service availability, and referral pathways may be more constrained. Fourth, gendered patterns of household access meant that adult men were underrepresented in screenings, which may have influenced observed risk distributions for certain conditions. Fifth, although qualitative coding was conducted independently by multiple researchers through iterative discussion and supervisory overview, formal interrater reliability was not calculated. Future studies may incorporate quantitative coding methods to enhance analytic reproducibility. Finally, although feasibility and usability were assessed, the study did not include formal measures of cost-effectiveness, health system readiness, or provider workload over time, which will be important considerations for scale-up.

Despite these limitations, the study provides important early evidence on the feasibility and acceptability of digitally enabled, community-led NCD screening within Pakistan’s primary healthcare system and offers actionable insights to inform future effectiveness–implementation research.

## Conclusion

This study demonstrates that community-based digital screening for non-communicable diseases (NCDs), delivered through Pakistan’s Lady Health Worker programme, is feasible and acceptable in urban and peri-urban settings. The NCD-SCAN application enabled structured household-level risk assessment across multiple NCD domains and was perceived by LHWs as enhancing their capacity to communicate risk and engage communities. In practice, the application could be embedded in routine lady health workers household visits as screening and counselling tool, while future iterations may incorporate linkages to primary care facilities, physician-supported triage and locally adapted multimedia educational content. To improve health outcomes, digital screening should be paired with effective referral pathways, adequately resourced and responsive health services, and financial protection mechanisms. Future hybrid effectiveness- implementation studies are needed to evaluate the cost effectiveness and inform sustainable scale up in low resource settings.

## Figures and Tables

**Table 1:** FGDs conducted in Karachi and Hyderabad

City / District	Area	Number of FGDs	Participants (n)
**Karachi**	District East	6	59
	District West	2	20
	**Subtotal**	**8**	**79**
**Hyderabad**	Hyderabad City	3	33
	Tando Jam	2	16
	**Subtotal**	**5**	**49**
**Total**	—	**13**	**128**

**Table 2:** Characteristics of Study Participants

Characteristic	Total
**Age (years), mean ± SD**	38.1 ± 14.3
**Gender**	
Male	392 (36.1)
Female	694 (63.8)
Other	1 (0.1)
**Education level**	
No formal education	247 (22.7)
Primary education	260 (23.9)
Secondary education	250 (23.0)
Higher secondary	218 (20.1)
Graduation or higher	112 (10.3)
**Language**	
Urdu	352 (32.4)
Sindhi	459 (42.2)
Punjabi	179 (16.5)
Balochi	2 (0.2)
Pashtu	24 (2.2)
Bengali	7 (0.6)
Saraiki	12 (1.1)
Other	51 (4.7)

**Table 3:** Overall NCD risk category

Low risk	246 (22.6)
Medium risk	516 (47.4)
High risk	325 (30.0)

**Table 4:** Characteristics of study participants by NCD risk status and city

	Karachi			Hyderabad		
	Risk status					
	Low 153	Medium 193	High 148	Low 93	Medium 332	High 178
**Age**	31.9 ± 9.8	41.5 ± 15.6	42.9 ± 15.2	30.1 ± 9.1	37.6 ± 13.4	40.6 ± 15.7
**Gender**						
**Male**	55 (35.9)	60 (31.1)	47 (31.8)	39 (41.9)	125 (38.8)	66 (37.1)
**Female**	97 (64.1)	133 (68.9)	101 (68.2)	54 (58.1)	197 (61.2)	112 (62.9)
**International Physical Activity Questionnaire Score (IPAQs)**						
**Low**	116 (75.8)	145 (75.1)	112 (75.7)	73 (78.5)	281 (87.3)	113 (63.5)
**Moderate**	28 (18.3)	36 (18.7)	19 (12.8)	9 (9.7)	22 (6.8)	39 (21.9)
**Vigorous**	9 (5.9)	12 (6.2)	17 (11.5)	11 (11.8)	19 (5.9)	26 (14.6)
**Hypertension**	23 (15.0)	38 (19.7)	69 (46.6)	29 (31.2)	85 (26.4)	82 (46.1)
**Diabetes (Rapid)**	0 (0.0)	62 (32.1)	50 (33.8)	0 (0.0)	80 (24.8)	48 (27.0)
**Angina (Rose)**	0 (0.0)	0 (0.0)	44 (29.7)	0 (0.0)	0 (0.0)	63 (35.4)
**Stroke (QVSFS)**	0 (0.0)	0 (0.0)	126 (85.1)	0 (0.0)	0 (0.0)	147 (82.6)
**Mental health (WHO5)**	0 (0.0)	157 (81.4)	89 (60.1)	0 (0.0)	299 (92.9)	121 (68.0)

Percentages are column-wise. Risk categories reflect screening outcomes rather than clinical diagnoses.

**Table 5:** CFIR-based summary of anticipated implementation determinants, including key barriers and facilitators identified during the study

CFIR Domain	CFIR Construct	Key findings	Barrier/Facilitator/Mixed
**Intervention Characteristics**	Adaptability	Multilingual interface (Urdu, Sindhi and English), offline functionality	Facilitator
	Design Quality and packaging	Integration of validated screening instruments, Urdu and Sindhi interface improved counselling and usability, LHW suggested addition of videos to explain disease	Facilitator
	Relative advantage	LHW were seen as trustworthy providers and the tool improved assessment and counselling LHW could not tell what problem someone had, can only guide and refer	Mixed
	Complexity	The comprehensive screening workflow required approximately 15–20 minutes per participant, and the addition of digital tasks increased perceived workload for LHWs.	Mixed
	Evidence Strength and Quality	Intervention used validated instruments, with successful completion of screenings with no data loss	Facilitator
	Cost (Indirect)	Use of the digital platform requires mobile data, may affect routine use	Barrier
**Outer settings**	Patient needs and resources	High unmet cardiometabolic risk and poor mental health were identified but Financial constraints can impede care-seeking decisions (eg. Following up on referrals)	Mixed
	External policies and incentives	The intervention aligns with the vision of Pakistan’s digital health framework (2022-2030), National action plan and expanding LHW mandate by the government. Lack of structured referral pathways and limited cross linkages.	Mixed
	External connectivity	Limited linkage between community screening and physicians and diagnostic services	Barrier
**Inner settings**	Structural characteristics	Implementation was done using LHW programme which is a large, mature and stable system operational for many decades. There were clearly defined catchment areas, clear role differentiation. Areas served were linguistically and ethnically diverse with central guidance but local execution	Facilitator
	Networks and communication	Effective communication between LHW and community members with effective engagement and counselling. However, the communication between LHW and physicians/referral pathways were limited	Mixed
	Culture	The LHW programme has a strong culture of community-based preventive care and LHW have a strong sense of professional identity and service ethic. Organizationally embedded role of LHW limits expectations beyond screening and referral (no role in decision making)	Facilitator
	Implementation climate	High demand for change due to NCD burden and strong compatibility with LHW role However, there are competing routine demands of LHW with added burden of digital workload were reported	Mixed
	Implementation readiness	District level support facilitates training and engagement. LHW training increased their confidence in community interaction. However, digital infrastructure including smartphones, internet reliability and support for referrals is incomplete	Mixed
**Characteristics of Individuals**	Knowledge, Beliefs and self-efficacy	LHWs recognized the increasing NCD burden and the need to address it Training helped increase their confidence in counselling	Facilitator
	Identification with organization and stage of change	LHWs showed strong identification with the LHW programme and saw themselves as trusted educators and first contact providers.	Facilitator
**Process**	Planning	Development involved multiple phases, intervention design was developed through focus group discussions. There was a structured training program	Facilitator
	Engaging	LHWs were key stakeholders and were actively engaged in local implementation. High uptake as Community members (innovation participants) were receptive due to trust in LHWs Limited engagement of physicians	Mixed
	Execution	High implementation fidelity with no data loss and offline synchronization	Facilitator
	Reflection and Evaluation	LHWs actively reflected by suggesting improvements in referral systems, teleconsultation and educational videos	Facilitator

**Table 6:** Brief thematic analysis of the post pilot feedback from lady health workers

Theme	Key Insights from Post-Pilot Feedback	Representative Observations
Training and Usability	Initial difficulty using the application improved with repeated training, peer support, and hands-on practice	“At first we couldn’t use it… after training it became easy”; peer learning and trainer support were frequently mentioned
Community Trust and Engagement	High trust in LHWs enabled strong participation, curiosity, and willingness to engage in screening	“If we asked one person, others gathered”; participants were eager to be included
Counselling and Behavior Change	The application facilitated real-time counselling and improved awareness of NCDs, with perceived adoption of preventive behaviors	LHWs reported advising on diet, salt intake, and physical activity; some noted personal and community-level behavior change
Operational Barriers in Field Implementation	Challenges included limited access to men during daytime visits, scheduling constraints, hesitation in responding to culturally sensitive questions, and initial skepticism about benefits	Evening/late visits required; participants asked, “what is the benefit?” reassuring maintenance of confidentiality improved response to sensitive questions such as alcohol intake

## Abbreviations

NCDs: Non-communicable diseases
LHWs: Lady Health Workers
LMICs: Low- and middle-income countries
mHealth: Mobile health
NCD-SCAN: Non-communicable Disease Screening Application
CFIR: Consolidated Framework for Implementation Research
FGDs: Focus group discussions
IDI: In-depth interviews
RAPID: Risk Assessment of Pakistani Individuals for Diabetes
QVSFS: Questionnaire for Verifying Stroke-Free Status
GATS: Global Adult Tobacco Survey
IPAQ: International Physical Activity Questionnaire
RFFQ: Refined Food Frequency Questionnaire
WHO-5: World Health Organization-Five Well-Being Index
SD: Standard deviation
IQR: Interquartile range
DHO: District Health Officer
DDHO: Deputy District Health Officer
SDG: Sustainable Development Goals
